# Pulse Oximetry Histogram Profiles Before and After Red Blood Cell Transfusion in Very Preterm Infants: A Prospective Observational Cohort

**DOI:** 10.3390/children13020167

**Published:** 2026-01-25

**Authors:** Nevra Çolak, Murat Konak, Saime Sündüs Uygun

**Affiliations:** 1 Department of Pediatrics, Selçuklu Medical Faculty, Selçuk University, Konya 42000, Türkiye; drnevracolak@gmail.com; 2 Division of Neonatology, Department of Pediatrics, Selçuklu Medical Faculty, Selçuk University, Konya 42000, Türkiye; uygunsaime@hotmail.com

**Keywords:** preterm infant, anemia of prematurity, red blood cell transfusion, pulse oximetry, oxygen saturation histogram, hypoxemia, hyperoxemia, bronchopulmonary dysplasia, retinopathy of prematurity

## Abstract

**Highlights:**

**What are the main findings?**

- Continuous SpO_2_ histogram monitoring showed only modest, non-significant shifts in hypoxemia, target-range oxygenation, and hyperoxemia over 72 h after RBC transfusion in very preterm infants.
- Despite a substantial hemoglobin increase after transfusion, oxygenation instability persisted and varied across higher-risk subgroups (BPD and birth weight < 1000 g).

**What are the implications of the main findings?**

- RBC transfusion should not be assumed to consistently normalize bedside oxygenation patterns; SpO_2_ histograms may provide a practical adjunct for assessing heterogeneous responses beyond hemoglobin values alone.
- Early post-transfusion histogram profiles may be useful for hypothesis generation and future risk-stratification studies for outcomes such as BPD and ROP, warranting validation in larger cohorts.

**Abstract:**

Background/Objectives: Red blood cell (RBC) transfusion is frequently used to treat anemia of prematurity, yet bedside metrics that capture its short-term impact on oxygenation stability are limited. We assessed whether pulse oximetry histogram-derived oxygen saturation (SpO_2_) exposure changes after transfusion and whether responses differ across clinical subgroups. Methods: This prospective observational cohort included preterm infants born <32 weeks’ gestation who received a standardized RBC transfusion (15 mL/kg). Continuous SpO_2_ histograms quantified the percentage of monitored time spent in hypoxemia (<85%), normoxemia (86–95%), and hyperoxemia (≥96%) during four intervals: 24 h pre-transfusion and 24, 48, and 72 h post-transfusion. Repeated-measures and subgroup analyses (BPD, sex, birth weight < 1000 g) were performed. Results: Thirty-three infants were analyzed (gestational age 29.4 ± 2.1 weeks; birth weight 1220.6 ± 316.9 g). Hemoglobin increased from 8.6 ± 1.1 to 11.7 ± 1.0 g/dL (*p* < 0.001). Cohort-level histogram shifts were modest: normoxemia increased from 68.4 ± 12.1% to 72.6 ± 11.4% at 24 h (*p* = 0.18), hypoxemia decreased from 10.3 ± 6.5% to 6.6 ± 4.8% (*p* = 0.09), and hyperoxemia remained stable (21.3 ± 9.2% to 20.8 ± 8.5%; *p* = 0.44). Infants with BPD and those <1000 g showed persistently higher hypoxemia and/or hyperoxemia at 72 h compared with counterparts. Exploratory ROC analyses showed modest discrimination of 24 h hypoxemia for ROP (AUC 0.71) and 72 h hyperoxemia for BPD (AUC 0.74). Conclusions: RBC transfusion corrected anemia but did not produce a consistent cohort-level improvement in SpO_2_ histogram stability. Histogram metrics may help characterize heterogeneous oxygenation responses and support hypothesis generation for individualized monitoring strategies.

## 1. Introduction

Preterm infants, particularly those born before 32 weeks of gestation, frequently experience oxygenation instability due to immaturity of respiratory control and cardiopulmonary physiology. Because cumulative exposure to both hypoxemia and hyperoxemia has been linked to major complications such as bronchopulmonary dysplasia (BPD) and retinopathy of prematurity (ROP), maintaining oxygen saturation (SpO_2_) within recommended target ranges is a central component of neonatal intensive care [1,2]. In parallel, anemia of prematurity is highly prevalent in this population, and red blood cell (RBC) transfusion remains among the most frequently performed interventions in very preterm infants [3]. Consistent with this, previous studies report that up to 60–90% of infants born at <32 weeks’ gestation receive at least one RBC transfusion during hospitalization [3].

Red blood cell (RBC) transfusion is commonly used to treat anemia of prematurity and improve oxygen-carrying capacity; however, the short-term physiological response to transfusion is not fully captured by hemoglobin increments alone [3]. Bedside assessment often relies on intermittent charted SpO_2_ values or summary averages, which may obscure clinically meaningful variability. Accordingly, accessible monitoring metrics that quantify cumulative oxygen exposure before and after transfusion are needed to characterize potential response patterns [4].

Near-infrared spectroscopy (NIRS) has been used to assess tissue-level oxygenation after transfusion, and recent evidence suggests that regional cerebral and splanchnic oxygenation indices can improve even when peripheral SpO_2_ changes little [5]. However, NIRS is not universally available and implementation varies across units. In contrast, pulse oximetry is ubiquitous in neonatal intensive care, and histogram-based monitoring summarizes continuous SpO_2_ distributions over prolonged periods by quantifying time spent in predefined bands of hypoxemia, target-range oxygenation, and hyperoxemia. This approach can capture cumulative exposure that may be missed by mean SpO_2_ values and has been used to support oxygen-management initiatives where narrow target ranges (often around 90–95%) are recommended [6,7,8,9,10].

Despite the widespread use of RBC transfusion, its short-term impact on cumulative oxygen exposure remains incompletely understood when assessed with continuous bedside monitoring. Therefore, we conducted a prospective observational cohort study of preterm infants born before 32 weeks’ gestation undergoing standardized RBC transfusion. Using continuous pulse oximetry histograms, we evaluated changes in time spent in hypoxemia (<85%), target-range oxygenation (86–95%), and hyperoxemia (≥96%) during the 24 h before transfusion and up to 72 h afterwards. We also assessed whether oxygenation profiles differed by clinically relevant subgroups (BPD, sex, and birth weight <1000 g) and explored associations between early post-transfusion histogram patterns and major neonatal outcomes. The present study addresses this gap by using pulse oximetry histogram profiles to quantify changes in hypoxemia, normoxemia, and hyperoxemia before and after RBC transfusion in very preterm infants.

## 2. Materials and Methods

### 2.1. Study Design and Setting

This prospective observational cohort study was conducted in the NICU of Selçuk University Faculty of Medicine (Konya, Türkiye) between January 2023 and December 2023. The primary research question of this study was whether red blood cell transfusion is associated with short-term changes in cumulative oxygen saturation exposure, as assessed by pulse oximetry histogram profiles, in very preterm infants. Secondary objectives included evaluating whether oxygenation responses differed across clinically relevant subgroups and exploring potential associations between early post-transfusion histogram patterns and neonatal outcomes.

### 2.2. Participants

Eligibility criteria were birth at <32 weeks’ gestation; admission during the study period; clinically indicated packed RBC transfusion; and complete histogram-based SpO_2_ data for all predefined windows. Exclusion criteria were major congenital anomalies, hemodynamic instability requiring invasive high-frequency ventilation before transfusion, culture-proven sepsis at the time of transfusion, or incomplete histogram data.

### 2.3. Transfusion Procedure

Leukoreduced and irradiated packed RBCs were transfused at 15 mL/kg over 3–4 h via peripheral venous access with continuous monitoring. Post-transfusion hemoglobin/hematocrit were obtained within 6 h as routine care.

### 2.4. Oxygen Saturation Monitoring and Histogram Extraction [11]

Continuous SpO_2_ monitoring was performed using Philips IntelliVue MP40 monitors (Philips Healthcare, Amsterdam, The Netherlands) and Nellcor™ OxiMax™ pulse oximeters (Medtronic/Covidien, Dublin, Ireland) (sampling frequency 1 Hz). Histogram-derived oxygenation data were extracted from bedside monitors. To minimize the influence of artefacts, histogram periods affected by obvious motion artefacts, signal loss, or probe dislodgement were identified using monitor quality indicators and bedside nursing documentation and were excluded from analysis. Continuous monitoring and standardized probe placement were used throughout the study period to improve data reliability.

### 2.5. Histogram-Based Oxygenation Metrics

Histograms were extracted for 24 h pre-transfusion and 24 h, 48 h, and 72 h post-transfusion. SpO_2_ exposure was categorized as hypoxemia (<85%), normoxemia (86–95%), and hyperoxemia (≥96%). The selected saturation thresholds were based on commonly used target ranges in preterm infants and aligned with major clinical trials and guideline recommendations [3,4]. During the study period, unit practice aimed to maintain SpO_2_ between 90% and 95% in infants receiving supplemental oxygen, with avoidance of prolonged exposure below 88% or above 95%. Accordingly, hypoxemia (<85%) was chosen to reflect clinically meaningful deep desaturation, normoxemia (86–95%) to represent the target range, and hyperoxemia (≥96%) to capture potentially excessive oxygen exposure.

### 2.6. Outcomes

The primary outcome was change in normoxemia (86–95%) across time windows. Secondary outcomes included hypoxemia and hyperoxemia exposure, narrow-band redistribution, hematologic response, and subgroup differences by sex, birth weight (<1000 g vs. ≥1000 g), and BPD. Exploratory ROC analyses evaluated associations between early post-transfusion histogram parameters and ROP, BPD, and survival.

Bronchopulmonary dysplasia (BPD) was defined according to standard criteria as the requirement for supplemental oxygen and/or respiratory support at 36 weeks’ postmenstrual age [12].

### 2.7. Statistical Analysis

Analyses were performed using SPSS v27.0. Changes over time were assessed with repeated-measures ANOVA or Friedman test. Between-group comparisons used independent-samples *t*-test or Mann–Whitney U test, as appropriate. ROC curves were used for exploratory outcome prediction; *p* < 0.05 was considered statistically significant. All subgroup analyses were unadjusted and exploratory in nature. Given the sample size, no multivariable adjustment was performed, and findings from subgroup and ROC analyses should be interpreted as hypothesis-generating.

## 3. Results

### 3.1. Participant Characteristics

Thirty-three preterm infants were included. Mean gestational age was 29.4 ± 2.1 weeks and mean birth weight was 1220.6 ± 316.9 g. At transfusion, corrected gestational age was 33.9 ± 3.2 weeks and mean weight was 1535.0 ± 512.9 g. Pre-transfusion hemoglobin and hematocrit were 8.6 ± 1.1 g/dL and 25.6 ± 3.1%, respectively (Table 1). No infant had culture-proven sepsis or hemodynamic instability at transfusion.

### 3.2. Hematologic Response

Hemoglobin increased from 8.6 ± 1.1 g/dL to 11.7 ± 1.0 g/dL (*p* < 0.001), and hematocrit increased from 25.6 ± 3.1% to 35.8 ± 3.4% (*p* < 0.001).

### 3.3. Primary Outcome: Histogram-Based Oxygenation Patterns

**Figure 1:**
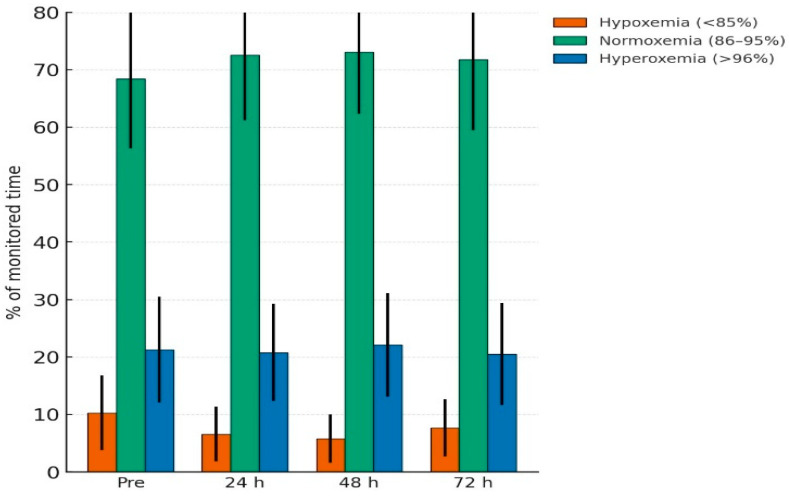
Histogram-based oxygen saturation distribution before and after transfusion. Mean percentage of monitored time spent in hypoxemia (<85%), normoxemia (86–95%), and hyperoxemia (≥96%) is shown at four time points: 24 h pre-transfusion and 24 h, 48 h, and 72 h post-transfusion. Error bars represent SD.

Cohort-level SpO_2_ histogram exposure showed modest, non-significant changes over 72 h. Normoxemia (86–95%) increased from 68.4 ± 12.1% pre-transfusion to 72.6 ± 11.4%, 73.1 ± 10.8%, and 71.8 ± 12.3% at 24 h, 48 h, and 72 h (*p* = 0.18). Hypoxemia (<85%) decreased from 10.3 ± 6.5% to 6.6 ± 4.8%, 5.8 ± 4.2%, and 7.7 ± 5.0% (*p* = 0.09). Hyperoxemia (≥96%) remained stable (*p* = 0.44). These primary histogram outcomes are presented in Table 2 and illustrated in Figure 1 (representative histogram distributions across timepoints).

#### Detailed SpO_2_ Band Distribution

SpO_2_ values were stratified into narrower bands (1–80%, 81–85%, 86–90%, 91–95%, 96–100%). After transfusion, there was a non-significant trend toward less time at 96–100% and more time at 91–95% (Table 3).

### 3.4. Subgroup Analyses

**Figure 2:**
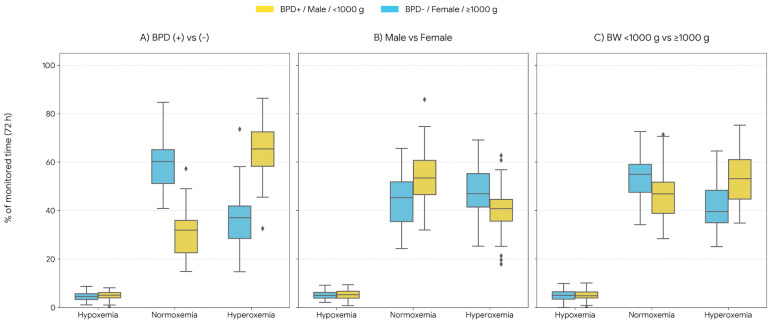
Subgroup comparison of oxygen saturation at 72 h after transfusion. Boxplots display hypoxemia (<85%), normoxemia (86–95%), and hyperoxemia (≥96%) stratified by BPD status, sex, and birth weight (<1000 g vs. ≥1000 g).

Subgroup analyses suggested heterogeneity in post-transfusion oxygenation profiles (Table 4; Figure 2). Male infants had higher hypoxemia exposure than females (*p*-between = 0.041). Infants with BPD had higher hypoxemia and hyperoxemia exposure than those without BPD (*p*-between = 0.023 and 0.037), with a within-group decrease in hypoxemia after transfusion (*p*-within = 0.049). Infants with birth weight <1000 g had higher hypoxemia exposure than infants ≥1000 g (*p*-between = 0.046) (Table 4). Given the limited sample size, subgroup comparisons were not powered to detect small-to-moderate differences and should be interpreted cautiously.

### 3.5. Exploratory ROC and Correlation Analyses

Exploratory ROC analyses suggested modest discrimination of 24 h hypoxemia for ROP (AUC 0.71 (95% CI 0.56–0.87); cut-off 7.5%; sensitivity 78.6%; specificity 65.2%; *p* = 0.038) and 72 h hyperoxemia for BPD (AUC 0.74 (95% CI 0.60–0.89); cut-off 22.0%; sensitivity 82.1%; specificity 68.4%; *p* = 0.026). Normoxemia at 48 h showed borderline discrimination for survival (AUC 0.68; *p* = 0.051). Correlation analyses showed: pre-transfusion FiO_2_ positively correlated with 24 h hyperoxemia (ρ = 0.42; *p* = 0.031); baseline hematocrit inversely correlated with 24 h hypoxemia (r = −0.36; *p* = 0.049); and mean SpO_2_ correlated with mechanical ventilation duration (r = 0.40; *p* = 0.028) (Table 5).

### 3.6. Short-Term Clinical Course and Safety

Within 48 h of transfusion, 5 infants (15%) were weaned to a lower level of respiratory support (*p* = 0.21). No acute transfusion-related adverse events, including apnea, bradycardia, or suspected transfusion reactions, were recorded during the monitoring period.

## 4. Discussion

This prospective observational cohort study evaluated the impact of red blood cell (RBC) transfusion on short-term oxygenation exposure in very preterm infants using histogram-based SpO_2_ monitoring, an approach increasingly used to quantify cumulative oxygen exposure beyond mean SpO_2_ or intermittent measurements [6,7,11]. Transfusion produced a clear hematologic response; however, whether hematologic correction translates into measurable improvement in oxygenation stability remains uncertain [4]. At the cohort level, histogram-derived SpO_2_ exposure changed only modestly over 72 h: time in the target-range band (86–95%) increased numerically, time in hypoxemia (<85%) decreased numerically, and hyperoxemia (≥96%) remained stable, with none of these shifts reaching statistical significance [4].

### 4.1. Comparison with Previous Findings

Prior studies assessing the respiratory effects of transfusion in preterm infants have yielded inconsistent results. Some authors have reported limited or negligible improvement in oxygen saturation following transfusion [12,13], whereas others have described transient reductions in desaturation events, particularly during the early post-transfusion period [14,15,16]. Our findings align more closely with studies demonstrating mild improvement without statistical significance, suggesting that transfusion alone may not substantially modify oxygenation patterns in an unselected preterm population.

Importantly, our use of histogram-based monitoring enables a more granular assessment of oxygen exposure than mean SpO_2_ values, which often fail to reflect true instability [6,7,11]. The observed trend toward reduced hypoxemia is clinically meaningful given the well-established associations between cumulative hypoxemia and BPD progression, impaired autonomic regulation, and increased risk of severe ROP [17,18]. Even modest reductions in hypoxemic exposure may therefore have physiological relevance.

### 4.2. Subgroup Heterogeneity and the Need for Individualized Transfusion Strategies

One of the most notable findings of this study was the heterogeneous response to transfusion among clinical subgroups. Male infants demonstrated greater variability in hypoxemia and hyperoxemia compared with females, reinforcing the well-described “male disadvantage” in neonatal respiratory outcomes [16,19,20,21]. Biological mechanisms such as delayed lung maturation, sex-specific hormonal regulation, and heightened inflammatory susceptibility may underlie these differences.

Infants with bronchopulmonary dysplasia (BPD) displayed persistently higher hyperoxemia and reduced post-transfusion stability. These findings are consistent with the pathophysiology of BPD, which includes disrupted alveolar development, ventilation–perfusion mismatch, and impaired oxygen handling [22,23]. The minimal improvement in normoxemia among infants with BPD suggests that increasing hemoglobin concentration alone may not overcome their underlying pulmonary limitations, echoing earlier reports emphasizing the limited role of transfusion in infants with chronic lung disease [24].

Extremely low birth weight (ELBW) infants (<1000 g) also exhibited more unstable oxygenation profiles, including higher hypoxemia at all time points. The vulnerability of this subgroup can be explained by immature respiratory control, higher oxygen extraction needs, and increased oxidative susceptibility—factors shown to influence responses to transfusion and oxygen therapy [17,25].

These subgroup patterns collectively support the emerging concept of precision-based transfusion strategies [3,26], where transfusion decisions incorporate factors such as baseline histogram stability, sex, lung disease severity, and birth weight rather than relying solely on hemoglobin thresholds [4].

### 4.3. Strength and Clinical Relevance of Histogram-Based Monitoring

A major methodological strength of this study is the use of histogram-derived oxygen saturation data. Manual or intermittent SpO_2_ documentation tends to overestimate time spent within target ranges and underestimate extreme saturation values, creating substantial observational bias [6]. Histogram monitoring avoids this by capturing continuous, high-frequency digital data, allowing clinicians to quantify true oxygen exposure—particularly time spent in hypoxemia and hyperoxemia, both of which are strongly associated with neonatal morbidity [17,24].

### 4.4. Physiological Interpretation and Relationship to Existing Evidence

The dissociation between hematologic correction and modest changes in peripheral SpO_2_ histograms is biologically plausible in a target-driven NICU environment. SpO_2_ is actively titrated within narrow limits through adjustments in FiO_2_ and respiratory support, which can constrain the extent to which saturation distributions shift after transfusion [17,24]. In this setting, transfusion may influence oxygen delivery more through increased arterial oxygen content and altered tissue extraction than through substantial changes in peripheral saturation.

This interpretation is consistent with evidence from near-infrared spectroscopy (NIRS), where regional cerebral and splanchnic oxygenation indices have been reported to improve following transfusion despite minimal changes in peripheral SpO_2_, including in a recent systematic review and meta-analysis [5]. Additionally, studies evaluating regional tissue oxygenation and desaturation patterns after transfusion support the concept that physiological effects may manifest at tissue level or in event dynamics rather than as large shifts in mean saturation distributions [16].

### 4.5. Exploratory Associations with Outcomes

Exploratory ROC analyses suggested modest discrimination of 24 h hypoxemia for ROP (AUC 0.71) and 72 h hyperoxemia for BPD (AUC 0.74), with borderline discrimination of 48 h normoxemia for survival (AUC 0.68). These signals are clinically plausible given established associations between oxygenation variability and adverse neonatal outcomes, but should be considered hypothesis-generating only [18,24]. In small cohorts, ROC-derived cut-offs are vulnerable to overfitting and require external validation before any clinical interpretation.

### 4.6. Clinical Implications

Our findings indicate that RBC transfusion should not be presumed to uniformly improve oxygenation stability in all very preterm infants. Histogram-derived metrics may be useful as an adjunctive monitoring tool—quantifying cumulative oxygen exposure and supporting structured review of oxygen titration practices—rather than as a standalone indicator of transfusion “success” [6,7,11]. The absence of an increase in hyperoxemia exposure at cohort level is reassuring within a target-driven unit policy; however, persistent instability in higher-risk subgroups suggests that hemoglobin correction alone may not normalize oxygen exposure patterns in infants with greater immaturity or lung disease [24]. An important clinical implication of this study is that recurrent oxygen desaturations alone should not be assumed to improve following RBC transfusion, nor should they automatically drive transfusion decisions. Although hemoglobin levels increased substantially, oxygenation instability often persisted, particularly in higher-risk subgroups. This finding is clinically relevant, as transfusion decisions in neonatal intensive care are frequently influenced by anxiety related to desaturation events among healthcare staff and families. Our results support a more cautious, physiologically informed approach in which desaturation patterns are interpreted in the broader context of underlying respiratory disease and overall clinical stability.

### 4.7. Strengths and Limitations

Strengths include the prospective design, predefined observation windows extending to 72 h, and use of histogram-derived SpO_2_ metrics capturing cumulative exposure across clinically relevant saturation bands [6,7,11]. Limitations include the observational single-center design without a non-transfused comparator, limited power for subgroup inference, and potential confounding by time-varying clinical factors that could not be fully standardized [13,14,22,27]. Peripheral SpO_2_ distributions also do not directly measure tissue oxygen delivery; concurrent NIRS or other physiological markers would strengthen mechanistic interpretation [5,16]. This study has several limitations. The single-center observational design and relatively small sample size limit generalizability and statistical power, particularly for subgroup analyses, increasing the risk of Type II error. In addition, the absence of multivariable adjustment limits causal inference. These limitations underscore the need for larger, multicenter studies to validate the observed oxygenation patterns and exploratory associations.

## 5. Conclusions

RBC transfusion in very preterm infants produced a robust hematologic response but only modest, non-significant cohort-level shifts in histogram-based SpO_2_ exposure over 72 h. Oxygenation response patterns were heterogeneous, with persistent instability in higher-risk infants such as those with BPD and extremely low birth weight. Histogram-derived SpO_2_ metrics are feasible for quantifying cumulative oxygen exposure and may support hypothesis generation regarding oxygenation phenotypes and outcome risk, but larger multicenter studies are needed before clinical thresholds can be recommended. Importantly, the lack of a consistent improvement in oxygenation stability following transfusion should be viewed as a clinically meaningful finding rather than a negative outcome. These results reinforce the concept that correction of anemia does not necessarily translate into immediate normalization of oxygen saturation patterns in very preterm infants.

## Figures and Tables

**Table 1:** Demographic and clinical characteristics of the study population (mean ± SD).

Variable	Value
Gestational Age (weeks)	29.4 ± 2.1
Corrected Gestational Age at Transfusion (weeks)	33.9 ± 3.2
Weight at Transfusion (g)	1535.0 ± 512.9
Weight Percentile at Transfusion	12.3 ± 18.6
Birth Weight (g)	1220.6 ± 316.9
Week of Transfusion (weeks)	4.5 ± 2.3
Hematocrit (%)	25.6 ± 3.1
Hemoglobin (g/dL)	8.6 ± 1.1

**Table 2:** Oxygen saturation distribution before and after transfusion (mean ± SD, % of monitored time).

SpO_2_ Range	Pre-Transfusion	24 h	48 h	72 h	*p*-Value
Normoxemia (86–95%)	68.4 ± 12.1	72.6 ± 11.4	73.1 ± 10.8	71.8 ± 12.3	0.18
Hyperoxemia (≥96%)	21.3 ± 9.2	20.8 ± 8.5	22.1 ± 9.0	20.5 ± 8.9	0.44
Hypoxemia (<85%)	10.3 ± 6.5	6.6 ± 4.8	5.8 ± 4.2	7.7 ± 5.0	0.09

**Table 3:** Detailed histogram-based SpO_2_ distribution across narrow saturation bands (mean ± SD, % of monitored time).

SpO_2_ Band	Pre	24 h	48 h	72 h	*p*-Value
96–100%	47.9 ± 27.1	46.7 ± 27.3	38.4 ± 23.1	43.9 ± 24.6	0.075
91–95%	36.9 ± 19.2	35.5 ± 17.3	43.2 ± 16.7	36.1 ± 14.6	0.087
86–90%	11.3 ± 9.9	12.5 ± 10.1	13.2 ± 10.7	13.9 ± 12.6	0.165
81–85%	2.5 ± 2.5	4.1 ± 5.8	4.2 ± 5.3	4.2 ± 4.8	0.214
1–80%	1.1 ± 1.2	1.6 ± 1.6	1.5 ± 1.8	1.4 ± 1.3	0.332

**Table 4:** Subgroup comparisons of SpO_2_ histogram outcomes after transfusion (mean ± SD, % of monitored time).

Subgroup	Outcome	Pre	24 h	48 h	72 h	*p*-Within	*p*-Between
Male vs. Female	Hypoxemia	11.2 ± 5.8	7.1 ± 4.3	6.0 ± 3.9	8.0 ± 4.6	0.062	0.041
Male vs. Female	Normoxemia	66.9 ± 11.5	71.4 ± 10.9	72.8 ± 10.2	70.6 ± 10.8	0.118	0.217
Male vs. Female	Hyperoxemia	21.9 ± 9.8	21.5 ± 9.1	21.2 ± 8.6	21.4 ± 8.8	0.384	0.492
BPD (+) vs. (−)	Hypoxemia	13.6 ± 6.2	8.5 ± 4.8	7.3 ± 4.1	9.2 ± 4.5	0.049	0.023
BPD (+) vs. (−)	Normoxemia	64.1 ± 12.4	70.3 ± 11.6	70.7 ± 10.8	69.0 ± 11.9	0.072	0.144
BPD (+) vs. (−)	Hyperoxemia	22.3 ± 8.9	21.2 ± 8.7	22.0 ± 9.3	21.8 ± 9.0	0.501	0.037
BW < 1000 g vs. ≥1000 g	Hypoxemia	12.7 ± 6.0	7.9 ± 4.4	6.8 ± 3.8	8.4 ± 4.2	0.053	0.046
BW < 1000 g vs. ≥1000 g	Normoxemia	65.7 ± 11.9	71.6 ± 11.1	72.0 ± 10.4	70.2 ± 10.9	0.099	0.182
BW < 1000 g vs. ≥1000 g	Hyperoxemia	21.6 ± 9.4	20.9 ± 8.8	21.7 ± 9.2	21.5 ± 8.9	0.427	0.058

**Table 5:** ROC and correlation analyses for post-transfusion oxygenation parameters and clinical outcomes.

Analysis	Variable/Comparison	AUC (95% CI)/r (ρ)	Cut-Off	Sensitivity (%)	Specificity (%)	*p*-Value
ROC	24 h hypoxemia → ROP	0.71 (0.56–0.87)	7.5%	78.6	65.2	0.038
ROC	72 h hyperoxemia → BPD	0.74 (0.60–0.89)	22.0%	82.1	68.4	0.026
ROC	48 h normoxemia → Survival	0.68 (0.53–0.83)	70.0%	70.8	66.7	0.051
Correlation	Pre-transfusionFiO_2_ → 24h hyperoxemia	ρ = 0.42	—	—	—	0.031
Correlation	Baseline Hct → 24 h hypoxemia	r = −0.36	—	—	—	0.049
Correlation	Mean SpO_2_ → ventilation duration	r = 0.40	—	—	—	0.028

## Data Availability

De-identified data are available from the corresponding author upon reasonable request, subject to institutional and legal requirements. All figures and tables are original and created by the authors; no previously published material is reproduced.

## References

[1] Villeneuve A., Arsenault V., Lacroix J., Tucci M. (2021). Neonatal red blood cell transfusion. Vox Sang..

[2] Leipälä J.A., Boldt T., Fellman V. (2004). Haemodynamic effects of erythrocyte transfusion in preterm infants. Eur. J. Pediatr..

[3] Deschmann E., Dame C., Sola-Visner M.C., Fustolo-Gunnink S.F., Guyatt G.H., Patel R.M., Stanworth S.J., Network N.T., New H., Lopriore E. (2024). Clinical practice guideline for red blood cell transfusion thresholds in very preterm neonates. JAMA Netw. Open.

[4] Saito-Benz M., Flanagan P., Berry M.J. (2020). Management of anaemia in pre-term infants. Br. J. Haematol..

[5] Zheng S.C., He S. (2025). The near-infrared spectroscopy to evaluate neonatal improvement after transfusion: A systematic review and meta-analysis. BMC Pediatr..

[6] Gentle S., El-Ferzli G., Winter L., Salas A.A., Philips J.B. (2020). Oxygen saturation histogram monitoring to reduce death or retinopathy of prematurity: A quality improvement initiative. J. Perinatol..

[7] Borenstein-Levin L., Konikoff L., Solimano A. (2020). Clinical quantification of SpO_2_ instability using a new histogram classification system: A clinical study. Pediatr. Res..

[8] Australia B.-I., Groups U.K.C. (2016). Outcomes of two trials of oxygen-saturation targets in preterm infants. N. Engl. J. Med..

[9] SUPPORT Study Group of the Eunice Kennedy Shriver NICHD Neonatal Research Network (2010). Target Ranges of Oxygen Saturation in Extremely Preterm Infants. N. Engl. J. Med..

[10] STOP-ROP Multicenter Study Group (2000). Supplemental therapeutic oxygen for prethreshold retinopathy of prematurity (STOP-ROP), a randomized, controlled trial. I: Primary outcomes. Pediatrics.

[11] Sur A., Paria A. (2021). Histogram analysis for bedside respiratory monitoring in not critically ill preterm neonates: A proposal for a new way to look at the monitoring data. Eur. J. Pediatr..

[12] Keir A., New H., Robitaille N., Crighton G., Wood E., Stanworth S. (2019). Approaches to understanding and interpreting the risks of red blood cell transfusion in neonates. Transfus. Med..

[13] Pandita A., Kumar A., Gupta G., Taligasalam V., Tewari V.V. (2020). Use of blood components in newborns. J. Neonatol..

[14] Ree I.M., Lopriore E. (2019). Updates in neonatal hematology: Causes, risk factors, and management of anemia and thrombocytopenia. Hematol./Oncol. Clin..

[15] Baysan M., Arbous M.S., Mik E.G., Juffermans N.P., van der Bom J.G. (2020). Study protocol and pilot results of an observational cohort study evaluating effect of red blood cell transfusion on oxygenation and mitochondrial oxygen tension in critically ill patients with anaemia: The INsufficient Oxygenation in the Intensive Care Unit (INOX ICU-2) study. BMJ Open.

[16] Seidel D., Bläser A., Gebauer C., Pulzer F., Thome U., Knüpfer M. (2013). Changes in regional tissue oxygenation saturation and desaturations after red blood cell transfusion in preterm infants. J. Perinatol..

[17] Atanasov S., Dippel C., Takoulegha D., Windhorst A., Schuler R., Strodthoff C., Frerichs I., Dreyhaupt J., Waitz M., Sohrabi K. (2023). Fluctuations in oxygen saturation during synchronized nasal intermittent positive pressure ventilation and nasal high-frequency oscillatory ventilation in very low birth weight infants: A randomized crossover trial. Neonatology.

[18] Sullivan B., Wallman-Stokes A., Isler J., Sahni R., Moorman J., Fairchild K., Lake D. (2018). Early pulse oximetry data improves prediction of death and adverse outcomes in a two-center cohort of very low birth weight infants. Am. J. Perinatol..

[19] Holditch-Davis D., Scher M., Schwartz T. (2004). Respiratory development in preterm infants. J. Perinatol..

[20] Migliori C., Braga M., Siragusa V., Villa M.C., Luzi L. (2023). The impact of gender medicine on neonatology: The disadvantage of being male: A narrative review. Ital. J. Pediatr..

[21] Peacock J.L., Marston L., Marlow N., Calvert S.A., Greenough A. (2012). Neonatal and infant outcome in boys and girls born very prematurely. Pediatr. Res..

[22] Collard K.J. (2014). Transfusion related morbidity in premature babies: Possible mechanisms and implications for practice. World J. Clin. Pediatr..

[23] Ambalavanan N., Deutsch G., Pryhuber G., Travers C.P., Willis K.A. (2026). The evolving pathophysiology of bronchopulmonary dysplasia. Physiol. Rev..

[24] Bancalari E., Claure N. (2018). Respiratory instability and hypoxemia episodes in preterm infants. Am. J. Perinatol..

[25] Dani C., Martelli E., Bertini G., Pezzati M., Rossetti M., Buonocore G., Paffetti P., Rubaltelli F.F. (2004). Effect of blood transfusions on oxidative stress in preterm infants. Arch. Dis. Child.-Fetal Neonatal Ed..

[26] Franz A.R., Engel C., Bassler D., Rüdiger M., Thome U.H., Maier R.F., Krägeloh-Mann I., Kron M., Essers J., Bührer C. (2020). Effects of liberal vs restrictive transfusion thresholds on survival and neurocognitive outcomes in extremely low-birth-weight infants: The ETTNO randomized clinical trial. JAMA.

[27] Keir A., Aziz K., McMillan D., Monterrosa L., Ojah C., Lee S., Shah P.S., Network C.N. (2015). Red blood cell transfusions at 21 days of age or older in previously transfusion-naive very preterm infants: Association with neonatal outcomes. Am. J. Perinatol..

